# Community Structure of Ammonia-Oxidizing Archaea and Ammonia-Oxidizing Bacteria in Soil Treated with the Insecticide Imidacloprid

**DOI:** 10.1155/2015/582938

**Published:** 2015-02-01

**Authors:** Mariusz Cycoń, Zofia Piotrowska-Seget

**Affiliations:** ^1^Department and Institute of Microbiology and Virology, School of Pharmacy with the Division of Laboratory Medicine, Medical University of Silesia, Jagiellońska 4, 41-200 Sosnowiec, Poland; ^2^Department of Microbiology, University of Silesia, Jagiellońska 28, 40-032 Katowice, Poland

## Abstract

The purpose of this experiment was to assess the effect of imidacloprid on the community structure of ammonia-oxidizing archaea (AOA) and ammonia-oxidizing bacteria (AOB) in soil using the denaturing gradient gel electrophoresis (DGGE) approach. Analysis showed that AOA and AOB community members were affected by the insecticide treatment. However, the calculation of the richness (*S*) and the Shannon-Wiener index (*H*) values for soil treated with the field rate (FR) dosage of imidacloprid (1 mg/kg soil) showed no changes in measured indices for the AOA and AOB community members. In turn, the 10∗FR dosage of insecticide (10 mg/kg soil) negatively affected the AOA community, which was confirmed by the decrease of the *S* and *H* values in comparison with the values obtained for the control soil. In the case of AOB community, an initial decline followed by the increase of the *S* and *H* values was obtained. Imidacloprid decreased the nitrification rate while the ammonification process was stimulated by the addition of imidacloprid. Changes in the community structure of AOA and AOB could be due to an increase in the concentration of N-NH_4_^+^, known as the most important factor which determines the contribution of these microorganisms to soil nitrification.

## 1. Introduction

Imidacloprid [1-(6-chloro-3-pyridylmethyl)-*N*-nitro-imidazolidin-2-ylideneamine] is a systemic insecticide used to control the insect pests and animal parasites [1, 2]. This insecticide has a high affinity to the nicotinic acetylcholine receptor of insects and acts as a neurotoxin [3]. Based on the results of many studies, the European Food Safety stated that neonicotinoids pose an unacceptably high risk to bees and that the industry-sponsored science upon which regulatory agencies claims of safety have relied may be flawed, or even deceptive [4].

Imidacloprid is characterised by a high persistence in soil with half-life up to 229 days, and its behaviour depends on different physicochemical and biological parameters such as organic matter, pH, temperature, crops, time, and microbial activity [5–7]. In our previous study [8], the results obtained with the phospholipid fatty acid (PLFA), the denaturing gradient gel electrophoresis (DGGE), and the community-level physiological profile (CLPP) approaches showed that imidacloprid induced significant changes in the composition of microbial communities and their metabolic activity. Moreover, the DGGE profiles and results of imidacloprid degradation study suggested the evolution of specific bacteria able to degrade this insecticide among autochthonous soil microorganisms [8]. With regard to the importance of nitrogen turnover for soil quality, presence of imidacloprid in soils may also affect the nontarget microorganisms responsible for these processes. Being one of them, nitrification plays a central role in the global nitrogen cycle of the environment and it is very sensitive to various contaminants including pesticides. As the first and rate-limiting step of nitrification in soil, oxidation of ammonia to nitrite is catalysed by the ammonia monooxygenase (AMO) [9, 10]. Previously, autotrophic ammonia-oxidizing bacteria (AOB) of beta- and gamma-proteobacteria were considered to be the most important contributors to ammonia oxidation. However, the recent development of molecular techniques has led to the discovery of* amoA* genes that encode the alpha-subunit of AMO, which raises questions about the role of AOB. Some studies have recently shown that ammonia-oxidizing archaea (AOA) that are distributed in soil ecosystems potentially represent the most important group of ammonia oxidizers [11–13]. The population sizes and community structure of AOB and AOA are shifting in response to temperature, pH, fertilizer levels, altitude, and contaminants including pesticides [9, 10, 14, 15]. Nevertheless, there is no information regarding the influence of imidacloprid on ammonia oxidizers structure in soil. Therefore, the aim of this study was to assess the impact of this insecticide on the genetic biodiversity of AOA and AOB using DGGE method. In addition, the nitrification rate and the number of culturable nitrifying bacteria based on measurement of nitrate concentration and plate count methods, respectively, were estimated.

## 2. Materials and Methods

### 2.1. Soil Sampling

A loamy sand soil collected from the top layer (A-horizon) to a maximum depth of 20 cm of grass-covered field located at the area of Pszczyna, Upper Silesia, in southern Poland (49°59′48′′N, 18°55′14′′E) was used. No plant protection products with imidacloprid or other pesticides have been used on the sampling place. Based on the FAO Soil Classification, collected soil was classified as Orthic Luvisol. For collected soil, physical and chemical parameters were assessed, and their values and methods of determination are shown in Table 1.

### 2.2. Soil Treatment

Certified standard of imidacloprid (99.8% chemical purity) was purchased from Sigma-Aldrich (Germany) and used for the contamination of soil. The performed experiment had a completely randomized block design that included three replications and the following treatments: control and two insecticide dosages (1 and 10 mg/kg soil) which corresponded to the recommended field rate (FR) of imidacloprid and 10 times the FR (10∗FR). Detailed information related to the experimental design and treatments is described in our previous study [8]. Samples of control and imidacloprid-treated soils were periodically removed (on days 1, 14, 28, and 56) for determination of the genetic biodiversity of AOA and AOB, the number of nitrifying bacteria, and the nitrification rate.

### 2.3. Determination of Nitrate and Ammonium Concentrations

The procedure of extraction of soil samples (10 g) with 100 mL of 0.1% K_2_SO_4_ for 24 h followed by the colorimetric assay of nitrate and ammonium concentrations in the filtrates was used. The intensity of the yellow colour that resulted from the reaction of nitrates with phenoldisulphonic acid (25% in conc. H_2_SO_4_) was measured, whereas ammonium was determined using the Berthelot reaction. The concentrations of both ions were estimated by reference to calibration curves and the blank values obtained, and the results were expressed as mg per kilogram of soil [16].

### 2.4. Enumeration of Nitrifying Bacteria

Soil samples (10 g) in 90 mL of 0.85% sterile NaCl medium (pH 7.0) were shaken at 120 rpm/min for 30 min. The viable counts of nitrifying bacteria were enumerated using the agar medium recommended by Aaronson [17] after incubation at 22°C for 10 days. Results were expressed as the log cfu (colony forming unit) per gram of dry soil.

### 2.5. Analysis of AOA and AOB Community Structure by PCR-DGGE Method

To extract the total DNA from soil samples, a GeneMATRIX Soil DNA Purification Kit (Eur_x_, Poland) was used according to the manufacturer's instruction. Next, the DNA was subjected to electrophoresis in a 1.0% (w/v) agarose gel followed by its quantification using the spectrophotometer method (Biophotometer, Eppendorf). Archaeal* amoA* genes were amplified using primers Arch-*amoA*F and arch-*amoA*R, while bacterial* amoA* genes were amplified by* amoA*-1F and* amoA*-2R (Table 2). For PCR reactions, a mixture that contained 1 × GoTaq Flexi Buffer (Promega), 2 mM MgCl_2_, 0.2 mM dNTP Mix (Promega), 0.5 *μ*M of each primer (Sigma-Aldrich), 0.2 *μ*g of DNA, and 1.5 U/*μ*L GoTaq DNA Polymerase (Promega) was used. The amplification procedure was performed using a PTC-118 Thermal Cycler (Bio-Rad, CA, USA) under the following conditions: (i) an initial denaturation step of 95°C for 3 min, (ii) 35 cycles of denaturation, annealing, and extension (95°C for 1 min followed by 53°C, AOA, or 55°C, AOB, for 1 min, with an extension step at 72°C for 1 min), and (iii) a final extension at 72°C for 7 min. After amplification, the products in the mixture were purified using a QIAquick PCR Purification Kit (Qiagen, USA) as described in the manufacturer's instruction.

The PCR products were analyzed in an 8% (w/v) polyacrylamide gel (37.5 : 1 acrylamide : bisacrylamide) composed of a linear denaturing gradient ranging from 30% to 55% and from 40% to 60% for AOA and AOB, respectively. Denaturant solutions were prepared by mixing the appropriate volumes of two (0–100%) denaturant stock solutions (7 mol/L urea and 40% v/v formamide). Electrophoresis was performed in a 1 × TAE buffer with a constant voltage of 80 V for 17 h at 60°C using a DCode Mutation Detection System (Bio-Rad, USA). The obtained gels were stained with ethidium bromide (0.5 mg/mL) followed by their analysis with Quantity One software (Bio-Rad, USA) to compare the AOA and AOB community structures between control and imidacloprid-treated soils. Phylogenic dendrograms that were based on the presence/absence of a band and band weighting (band density) analyses were constructed by applying the Dice coefficient and the unweighted pair-group method using the arithmetic averages (UPGMA). Richness (*S*) values were calculated as the number of DNA bands detected in the respective line of the DGGE profile, while the Shannon-Wiener index (*H*) and evenness (*E*_*H*_) values were calculated according to the equations *H* = −∑*p*_*i*_(ln⁡⁡*p*_*i*_) and *E*_*H*_ = *H*/*H*_max⁡_ = *H*/ln⁡⁡*S*, respectively, where *p*_*i*_ is the ratio between the specific band intensity and the total intensity of all bands and *S* is the total number of bands in each sample.

### 2.6. Statistical Analyses

To determine the percentage of the variation attributable to the tested factors, that is, treatment and incubation time, a two-way analysis of variance (ANOVA) for the obtained results was applied. The statistical significance of differences in the data that was measured was assessed by a post hoc comparison of the means using the least significant differences (LSD) test. The obtained results were also subjected to principal component analysis (PCA). To determine the correlations between measured parameters, Pearson's correlation coefficient was also calculated. All analyses were performed using the Statistica 10.0 PL software package.

## 3. Results

### 3.1. Concentration of N-NO_3_^−^ and N-NH_4_^+^

**Figure 1:**
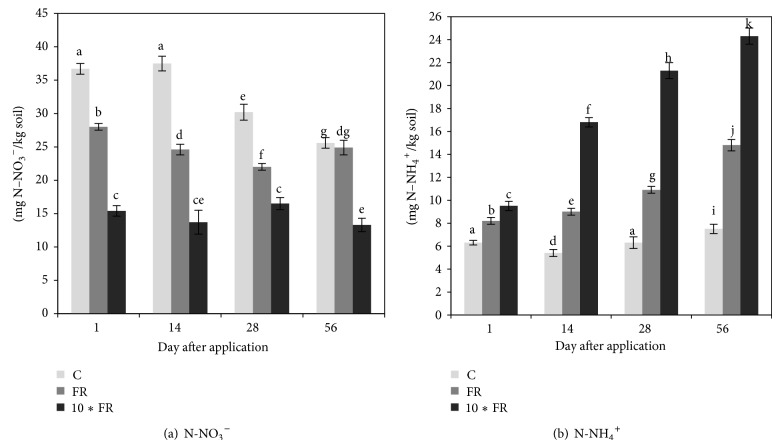
The concentration (mg/kg soil) of N-NO_3_^−^ (a) and N-NH_4_^+^ (b) in soil treated with imidacloprid (C: control; FR: 1 mg/kg soil; 10∗FR: 10 mg/kg soil). The data presented are the means and standard deviations of three replicates. The different letters indicate significant differences (*P* < 0.05, LSD test), considering the effects of the pesticide dosage and time.

The obtained data revealed that the application of the insecticide imidacloprid had a significant effect on nitrogen transformation in soil. Imidacloprid at both dosages caused a significant (*P* < 0.05) decrease (by 25–65%) in N-NO_3_^−^ concentrations and this effect was observed throughout the whole incubation period (Figure 1(a)). In contrast, both imidacloprid dosages caused a significant (*P* < 0.05) increase of the N-NH_4_^+^ concentration in soils over the experimental period. For example, the concentration of N-NH_4_^+^ in the 10∗FR-treated soil was several times higher than in the control soil on days 14, 28, and 56 (Figure 1(b)). The statistical analysis indicated that concentrations of both ions were significantly affected by the treatment (*P* < 0.001) and the incubation time (*P* < 0.001). In addition, the interaction between these factors was also significant (*P* < 0.001). For both ions, the treatment effect explained most of the variance (83% for N-NO_3_^−^ and 64% for N-NH_4_^+^) (Table 3). Values of Pearson's correlation coefficient (*r*) also indicated that the concentration of N-NO_3_^−^ was negatively correlated with N-NH_4_^+^ (−0.776, *P* < 0.001) and *E*_*H*_-AOA (−0.478, *P* = 0.003) while being positively correlated with the number of culturable nitrifying bacteria (0.866, *P* < 0.001), *H*-AOA (0.663, *P* < 0.001), *S*-AOA (0.727, *P* < 0.001), *H*-AOB (0.417, *P* = 0.011), and *S*-AOB (0.377, *P* < 0.023). In contrast, N-NH_4_^+^ was negatively correlated with the number of culturable nitrifying bacteria, *H*-AOA, and *S*-AOA (Table 4).

### 3.2. Number of Culturable Nitrifying Bacteria

**Figure 2:**
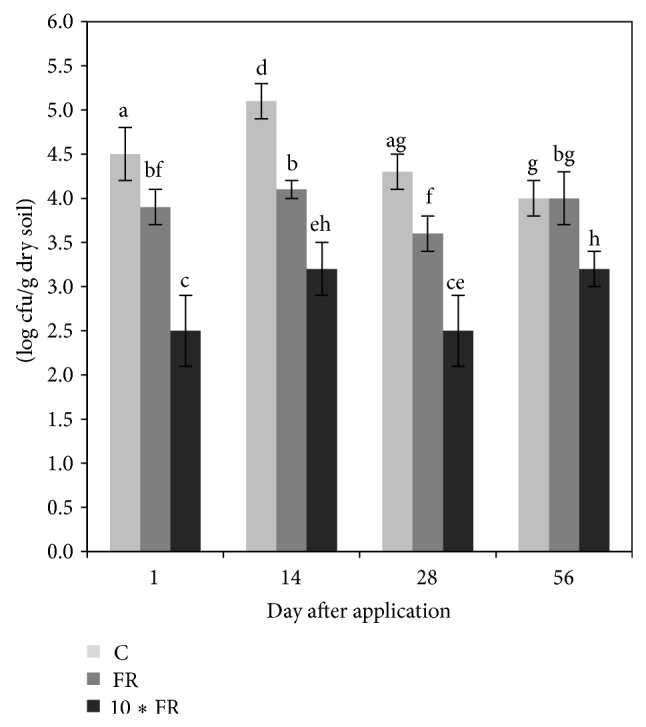
The number of nitrifying bacteria (log cfu/g dry soil) in soil treated with imidacloprid (C: control; FR: 1 mg/kg soil; 10∗FR: 10 mg/kg soil). The data presented are the means and standard deviations of three replicates. Different letters indicate significant differences (*P* < 0.05, LSD test), considering the effects of the pesticide dosage and time.

The plate count results showed that the application of imidacloprid negatively affected the number of culturable nitrifying bacteria. In the case of lower dosage of imidacloprid, this effect lasted up to 28 days. In contrast, in soil treated with imidacloprid at the 10∗FR dosage a negative effect was observed during the whole experimental period, and the number of culturable nitrifying bacteria was significantly (*P* < 0.05) lower (one or two orders of magnitude) than that in the control samples (Figure 2). The ANOVA showed that the number of culturable nitrifying bacteria was significantly affected by the treatment (*P* < 0.001) and the incubation time (*P* < 0.001). In addition, the interaction between these factors was also significant (*P* = 0.004). The treatment effect explained most of the variance (74%) whereas the incubation time and the interaction between both factors accounted for only 10% and 8% of the variance, respectively (Table 3). Values of Pearson's correlation coefficient (*r*) also indicated that the number of culturable nitrifying bacteria was positively correlated with *H*-AOA (0.717, *P* < 0.001), *S*-AOA (0.731, *P* < 0.001), *H*-AOB (0.452, *P* = 0.006), and *S*-AOB (0.436, *P* = 0.008) (Table 4).

### 3.3. Community Structure of Ammonia-Oxidizing Microorganisms

#### 3.3.1. Ammonia-Oxidizing Archaea (AOA)

**Figure 3:**
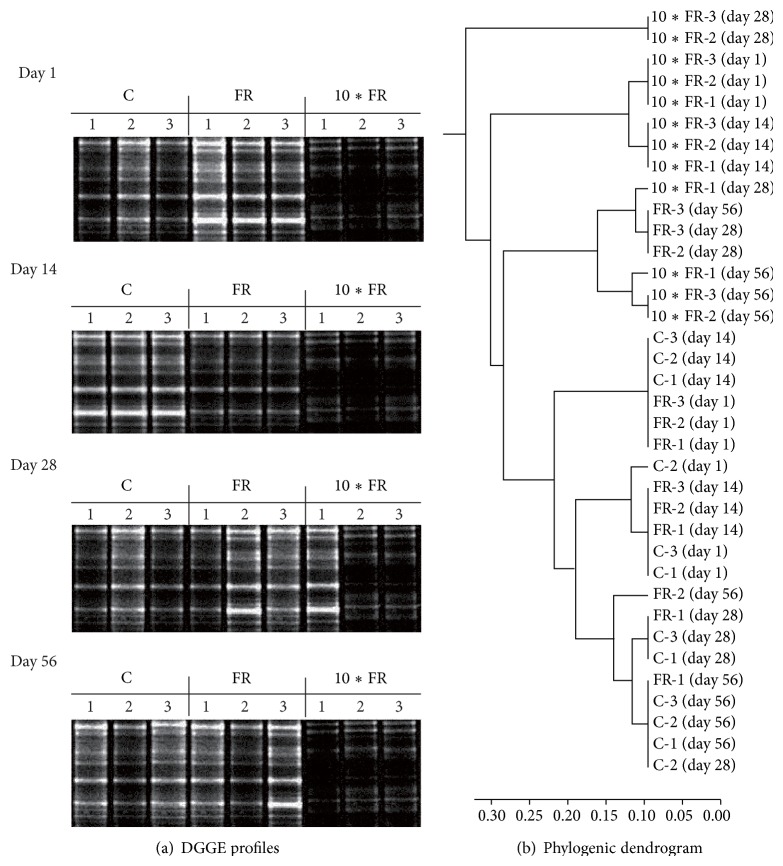
DGGE profiles (a) and phylogenic dendrogram (b) for PCR-amplified archaeal* amoA* gene for soil treated with imidacloprid (C: control; FR: 1 mg/kg soil; 10∗FR: 10 mg/kg soil).

**Figure 4:**
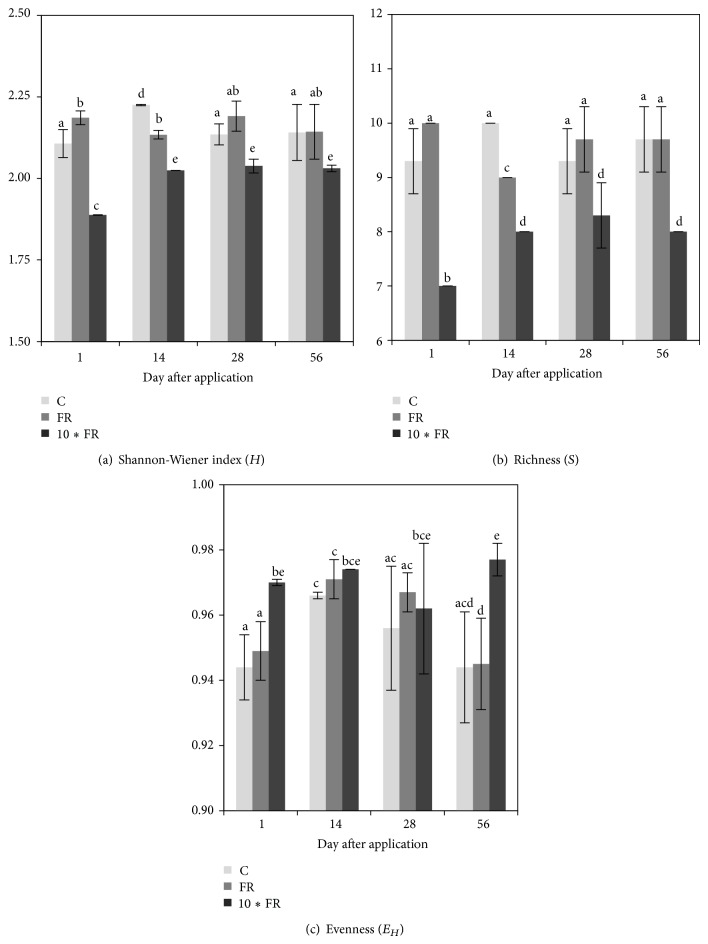
The diversity indices for ammonia-oxidizing archaea (AOA) in soil treated with imidacloprid (C: control; FR: 1 mg/kg soil; 10∗FR: 10 mg/kg soil). The data presented are the means and standard deviations of three replicates. Different letters (within each index) indicate significant differences (*P* < 0.05, LSD test), considering the effects of the pesticide dosage and time.

The obtained DGGE patterns showed that both dosages of imidacloprid caused changes in the structure of the AOA community during the experimental period (Figure 3(a)). The dosage of insecticide was the main factor that grouped the treatments, regardless of the time elapsed as indicated by the performed cluster analysis (Figure 3(b)). Despite the differences in the DGGE profiles between the imidacloprid-treated and control soil, the calculation of the Shannon-Wiener index (*H*) (Figure 4(a)) and the richness (*S*) (Figure 4(b)) values for soil treated with the field rate (FR) dosage of imidacloprid (1 mg/kg soil) showed no changes in measured indices for the AOA community members. In turn, the 10∗FR dosage of insecticide (10 mg/kg soil) negatively affected the AOA community, which was confirmed by the decrease of the *H* and *S* values in comparison with the values obtained for the control soil (Figures 4(a) and 4(b)).

The ANOVA analysis revealed that the *H* index for AOA was significantly affected by the treatment (*P* < 0.001) and the incubation time (*P* = 0.009). In addition, the interaction between these factors was also significant (*P* = 0.002). The treatment effect explained 64% of the variance, whereas time accounted for only 8% of the variance. The interactions between these factors explained a further 16% (Table 3). The richness value (*S*) for AOA was also significantly affected by the treatment (*P* < 0.001) as well as by the interaction between treatment and time (*P* = 0.002). The treatment effect explained most of the variance (72%) and the interactions between these factors explained a further 14% (Table 3). The two-way ANOVA also indicated that the incubation time and the interactions between treatment and time were factors which did not significantly affect the *E*_*H*_ value for AOA during the experiment. However, the treatment effect significantly (*P* = 0.004) affected the measured parameter, and it explained most of the variance (24%) (Table 3).

#### 3.3.2. Ammonia-Oxidizing Bacteria (AOB)

**Figure 5:**
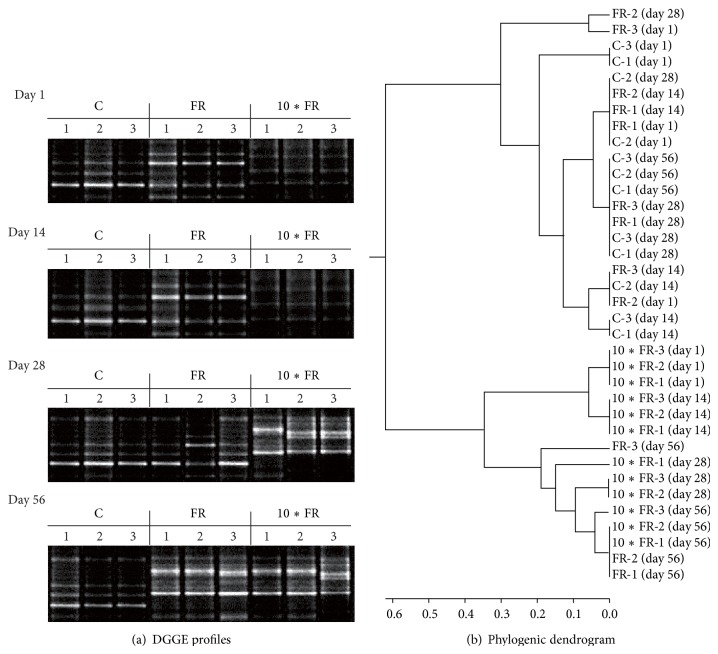
DGGE profiles (a) and phylogenic dendrogram (b) for PCR-amplified bacterial* amoA* gene for soil treated with imidacloprid (C: control; FR: 1 mg/kg soil; 10∗FR: 10 mg/kg soil).

**Figure 6:**
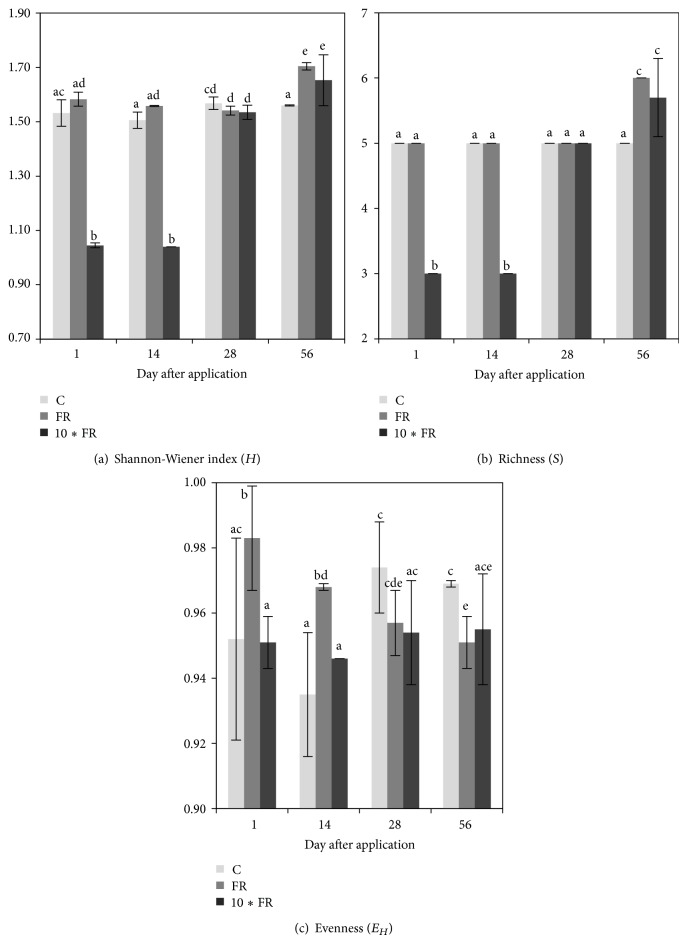
The diversity indices for ammonia-oxidizing bacteria (AOB) in soil treated with imidacloprid (C: control; FR: 1 mg/kg soil; 10∗FR: 10 mg/kg soil). The data presented are the means and standard deviations of three replicates. Different letters (within each index) indicate significant differences (*P* < 0.05, LSD test), considering the effects of the pesticide dosage and time.

The PCR-DGGE profiles of soil AOB community under imidacloprid stress also showed that there were significant changes in DGGE fingerprints among both treatments and control soil (Figure 5(a)). The dosage of insecticide was the main factor that grouped the treatments, regardless of the time elapsed as indicated by the performed cluster analysis (Figure 5(b)). Cluster analysis generally showed that imidacloprid dosage was the main factor grouping the treatments, regardless of the time elapsed (Figure 5(b)). Despite the differences in the DGGE profiles between the imidacloprid-treated and control soil, the calculation of the Shannon-Wiener index (*H*) (Figure 6(a)) and the richness (*S*) (Figure 6(b)) values for soil treated with the field rate (FR) dosage of imidacloprid (1 mg/kg soil) showed no changes in measured indices for the AOB community members on days 1, 14, and 28. In contrast, the values of both indices (*H* and *S*) were significantly (*P* < 0.005) higher on day 56 in comparison with the values obtained for the control soil. In the case of the 10∗FR dosage of insecticide, an initial decline (on days 1 and 14) followed by the increase (on day 56) of the *S* and *H* values was obtained for AOB community (Figures 6(a) and 6(b)).

The statistical analysis revealed that the *H* index for AOB was significantly (*P* < 0.001) affected by the treatment and the incubation time. In addition, the interaction between these factors was also significant. Both factors and interaction between them explained more or less equal (30%–35%) variance (Table 3). The richness value (*S*) for AOB was also significantly (*P* < 0.001) affected by the treatment and the time as well as by the interaction between both factors. The interaction between considered factors explained most of the variance (36%). The treatment and the time explained further 24% and 34% of the variance, respectively (Table 3). The ANOVA also indicated that the interaction between treatment and time was the only factor significantly affecting the *E*_*H*_ value during the experiment (Table 3).

## 4. Discussion

**Figure 7:**
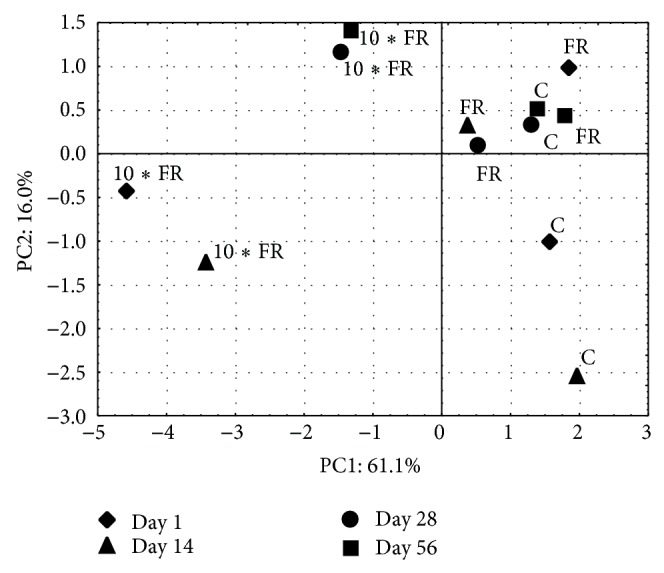
A principal component plot generated from the data (nitrification rate, number of nitrifying bacteria, and DGGE) obtained for soil treated with imidacloprid (C: control; FR: 1 mg/kg soil; 10∗FR: 10 mg/kg soil).

The impact of imidacloprid on the structure of ammonia-oxidizing microorganisms was assessed via PCR-DGGE. Complex fingerprints for both microbial groups (AOA and AOB) were obtained and multivariate statistical analysis was used to assess the effects of insecticide and sampling time on their community structure. The two-way ANOVA indicated that measured parameters were significantly (*P* < 0.05) affected by the treatment and the incubation time, and moreover the interaction between these factors was also significant. In general, the treatment effect explained most of the variance (Table 3). Moreover, the PCA clearly separated measured parameters especially in 10∗FR-treated and control soil samples. The principal components PC1 and PC2 accounted for 61.1% and 16% of this variation, respectively (Figure 7). Analysis of correlation among tested parameters showed that PC1 values correlated mainly with the number of culturable nitrifying bacteria as well as with the biodiversity of AOA and AOB community members, whereas PC2 values correlated mainly with the biodiversity of AOB.

Imidacloprid negatively affected the concentration of N-NO_3_^−^ in soil, whereas the concentration of N-NH_4_^+^ has increased during the experimental period. Many authors confirmed that nitrification process is more sensitive to pesticides than ammonification. For example, Martinez-Toledo et al. [18], studying the effect of methylpyrimifos on soil microflora, revealed that this insecticide significantly decreased the nitrification rate in agricultural soil. Similarly, other insecticides (e.g., diazinon, teflubenzuron, lambda-cyhalothrin, phorate, carbofuran, and fenvalerate) were found to be the factors responsible for the significant decrease of N-NO_3_^−^ in soil [19–22]. This phenomenon observed for the imidacloprid-treated soil can be explained by the fact that archaea and/or bacteria involved in ammonia oxidizing might be killed by the insecticide and the transformation of N-NH_4_^+^ into N-NO_3_^−^ was stopped in the soil. This fact may also be confirmed by the plate count data which showed the decrease of the number of culturable nitrifying bacteria during the experimental period. It is also a high probability that the degradation process of imidacloprid could also have resulted in the production of N-NH_4_^+^. Moreover, the results of some studies have shown that pesticides stimulate ammonification by killing a remaining sensitive part of soil microorganisms that are subjected to mineralisation processes, consequently increasing the ammonium concentration in soils [23].

The DGGE analysis showed that AOA were more sensitive to imidacloprid than AOB. Moreover, there were differences between profiles obtained for the imidacloprid-treated and control soils manifested by the disappearance of some bands (for AOA) or the appearance of several new bands (for AOB), especially in soil samples treated with the 10∗FR dosage. The richness (*S*) and genetic diversity (*H*) values for AOA were significantly lower in comparison with the values obtained for the control soil, whereas for AOB the diversity indices changed from decreasing at the initial stage to increasing at the end of the experimental period, suggesting that imidacloprid may stimulate the increase of AOB. The observed phenomenon suggests that sensitive species among ammonia-oxidizing microorganisms were replaced by microorganisms characterised by a higher tolerance to this insecticide and/or the ability to degrade imidacloprid. The result of these changes might be the increase of the numbers of specific microorganisms and the decrease in the overall richness (*S*) and the diversity (*H*) of AOA and/or AOB community members. Several studies have demonstrated that the excessive use of pesticides has raised concerns regarding the toxic effects on nontargeting microorganisms. Increasing evidence has indicated that pesticides change the community structure of AOA and/or AOB and decrease the nitrification rate in soil. For example, using DGGE, Li et al. [24] demonstrated that acetochlor had a negative effect on AOB diversity in soil. Pampulha and Oliveira [25] indicated that the presence of bromoxynil and prosulfuron strongly inhibited the growth of AOB in sandy soil. Gigliotti and Allievi [26] described that cinosulfuron and bensulfuron, at the normal field application rate and at a 100-fold higher rate, decreased the nitrification activity in agricultural soil.

Our results also suggest that observed changes in the community structure of AOA and AOB could be due to an increase in the concentration of N-NH_4_^+^. In some previous studies on agricultural soils, long-term N fertilizer application seemed to stimulate the growth of AOB [13, 27]. This could be explained by a stimulatory effect of high N-NH_4_^+^ concentration on the AOB diversity and richness. On the contrary, the diversity and richness indices for AOA (Figure 4) showed a declining trend by increasing N-NH_4_^+^ concentration during the experimental period (Figure 1(b)). Similar results have been reported by Di et al. [28], who observed that AOB and AOA had different growth patterns under contrasting soil N conditions. In addition, analysis of values of Pearson's correlation coefficient (*r*) revealed significant correlations (*P* < 0.05) between the values of diversity and richness indices for AOA and AOB and N-NH_4_^+^ concentration (Table 4). These results indicated that N-NH_4_^+^ concentration may be the most important factor determining the contribution of these microorganisms to soil nitrification. Moreover, as found in different ecosystems, the AOA* amoA* copy numbers were more abundant than AOB [29–31]. Therefore, our results also provide further evidences to support the hypothesis that AOB growth is favoured by high concentration of N-NH_4_^+^, whereas AOA have a preference for low N-NH_4_^+^ concentration in soil [32]. Although the diversity and richness of AOB community members were higher after application of 10∗FR dosage of imidacloprid, nitrification rate was lower in comparison with the control soil as indicated by the decrease of N-NO_3_^−^. These results and the simultaneous decrease in the diversity and richness of AOA community members may further support hypothesis that AOA rather than AOB control nitrification [15]. However, some authors showed that soil nitrification was significantly correlated with the abundance of bacterial* amoA* genes [30, 31]. Such differences in contributions of AOB and AOA may be due to different factors, among which the most important is probably the concentration of N-NH_4_^+^ [15, 33].

## 5. Conclusions

Based on the obtained results, we concluded that the application of imidacloprid changed the structure of ammonia-oxidizing microorganisms. Insecticide significantly increased the diversity and richness of AOB but suppressed the AOA community member. Moreover, imidacloprid negatively affected the nitrification rate in soil, which was confirmed by the decrease of N-NO_3_^−^ concentration during the experiment, whereas the concentration of N-NH_4_^+^ in soil was higher than in the control. Taking into consideration the preference of AOA for low N-NH_4_^+^ concentration, our results showed that the contribution of AOA to nitrification process may be more significant than that by AOB under low concentration of N-NH_4_^+^ in soil. Ammonia-oxidizing microorganisms are the sensitive microbial indicators and may be used to evaluate the impact of pesticides on soil quality; however, the effects of these chemicals on nitrification process appear to be species specific.

## Figures and Tables

**Table 1:** Characteristics of the soil used in the experiment.

Parameter	Value	Method of determination	Reference
Origin	Pszczyna, Poland	—	—
Sand (2000–50 *μ*m) (%)	86.0 ± 2.7	Sedimentation and sieving method	ISO 11277:2009
Silt (<50–2 *μ*m) (%)	11.0 ± 2.4	Sedimentation and sieving method	ISO 11277:2009
Clay (<2 *μ*m) (%)	3.0 ± 0.5	Sedimentation and sieving method	ISO 11277:2009
Density g/cm^3^	1.2 ± 0.2	Core method	ISO 11272:1998
pH_(in water)_ (1 : 5)	6.6 ± 0.3	Measurement with glass electrode	ISO 10390:2005
Cation exchange capacity (CEC) (cmol+/kg)	12.9 ± 1.7	Modified Gillman method	ISO 11260:1994
Water holding capacity (WHC) (%)	32.4 ± 2.8	Gravimetric method	ISO 14239:1997
C_org_ (%)	1.0 ± 0.2	Oxidation in the presence of H_2_SO_4_	ISO 14235:1998
N_tot_ (%)	0.09 ± 0.03	Modified Kjeldahl method	ISO 11261:1995
Microbial biomass (mg/kg dry weight)	668.0 ± 34.2	Substrate-induced respiration (SIR)	ISO 14240-1:1997

The values are the means of three replicates with the standard deviation, which was within 5% of the mean.

**Table 2:** Primers used for molecular analyses in this study.

Targets	Primers	Sequence (5′-3′)	Length of amplicon (bp)	References
AOA	Arch-*amoA*F	STA ATG GTC TGG CTT AGA CG	635	[34]
	arch-*amoA*R	GCG GCC ATC CAT CTG TAT GT		

AOB	*amoA*-1F	GGG GTT TCT ACT GGT GGT	491	[35]
	*amoA*-2R	CCC CTC KGS AAA GCC TTC TTC		

AOA: ammonia-oxidizing archaea; AOB: ammonia-oxidizing bacteria. Forty-base pair GC-clamp, CGC CCG CCG CGC GCG GCG GGC GGG GCG GGG GCA CGG GGG C [36], was attached to the 5′ end of the forward primers.

**Table 3:** The two-way ANOVA analysis for the measured parameters.

Parameter	Source of variation	df	Sum of squares	Mean squares	Variance explained (%)	*F*	*P*
Concentration of ions							
N-NO_3_^−^	Treatment	2	1908.79	954.40	83	989.3	**P** ** < 0.001**
	Time	3	158.20	52.73	7	54.6	**P** ** < 0.001**
	Treatment × time	6	204.12	34.02	9	35.3	**P** ** < 0.001**
N-NH_4_^+^	Treatment	2	819.2	409.6	64	2275.5	**P** ** < 0.001**
	Time	3	282.2	94.1	22	522.6	**P** ** < 0.001**
	Treatment × time	6	13.9	28.9	13	161.1	**P** ** < 0.001**
Number of nitrifying bacteria	Treatment	2	15.65	7.83	74	124.7	**P** ** < 0.001**
	Time	3	2.23	0.74	10	11.8	**P** ** < 0.001**
	Treatment × time	6	1.63	0.27	8	4.3	**P** ** = 0.004**
AOA-DGGE analysis							
*H*	Treatment	2	0.21	0.11	64	61.2	**P** ** < 0.001**
	Time	3	0.02	0.01	8	4.8	**P** ** = 0.009**
	Treatment × time	6	0.05	0.001	16	5.1	**P** ** = 0.002**
*S*	Treatment	2	24.50	12.25	72	73.5	**P** ** < 0.001**
	Time	3	0.66	0.22	2	1.3	*P* = 0.286
	Treatment × time	6	4.83	0.81	14	4.8	**P** ** = 0.002**
*E*_*H*_	Treatment	2	0.002	0.0009	24	7.1	**P** ** = 0.004**
	Time	3	0.001	0.0004	15	3.0	*P* = 0.052
	Treatment × time	6	0.002	0.0003	20	2.0	*P* = 0.112
AOB-DGGE analysis							
*H*	Treatment	2	0.52	0.26	34	214.6	**P** ** < 0.001**
	Time	3	0.46	0.15	30	126.3	**P** ** < 0.001**
	Treatment × time	6	0.53	0.09	35	72.9	**P** ** < 0.001**
*S*	Treatment	2	7.72	3.86	28	139.0	**P** ** < 0.001**
	Time	3	9.42	3.14	34	113.0	**P** ** < 0.001**
	Treatment × time	6	9.83	1.63	36	59.0	**P** ** < 0.001**
*E*_*H*_	Treatment	2	0.0012	0.0005	10	2.5	*P* = 0.102
	Time	3	0.0009	0.0003	8	1.4	*P* = 0.267
	Treatment × time	6	0.0039	0.0007	36	3.1	**P** ** = 0.023**

AOA: ammonia-oxidizing archaea; AOB: ammonia-oxidizing bacteria; *H:* Shannon-Wiener index; *S*: richness; *E*_*H*_: evenness. The effects in bold are significant at *P* < 0.05.

**Table 4:** Values of Pearson's correlation coefficient (*r*) for correlation among measured microbial parameters.

	N-NO_3_^−^	N-NH_4_^+^	Number	*H*-AOA	*S*-AOA	*E*_*H*_-AOA	*H*-AOB	*S*-AOB	*E*_*H*_-AOB	PC1	PC2
N-NO_3_^−^	1										
N-NH_4_^+^	**−0.776** **P** ** < 0.001**	1									
Number	**0.866** **P** ** < 0.001**	**−0.633** **P** ** < 0.001**	1								
*H*-AOA	**0.663** **P** ** < 0.001**	**−0.414** **P** ** = 0.012**	**0.717** **P** ** < 0.001**	1							
*S*-AOA	**0.727** **P** ** < 0.001**	**−0.488** **P** ** = 0.003**	**0.731** **P** ** < 0.001**	**0.952** **P** ** < 0.001**	1						
*E*_*H*_-AOA	**−0.478** **P** ** = 0.003**	**0.357** **P** ** = 0.033**	**−0.358** **P** ** = 0.032**	−0.244 *P* = 0.152	**−0.498** **P** ** = 0.002**	1					
*H*-AOB	**0.417** **P** ** = 0.011**	0.016 *P* = 0.925	**0.452** **P** ** = 0.006**	**0.601** **P** ** < 0.001**	**0.618** **P** ** < 0.001**	−0.324 *P* = 0.054	1				
*S*-AOB	**0.377** **P** ** = 0.023**	0.089 *P* = 0.607	**0.436** **P** ** = 0.008**	**0.571** **P** ** < 0.001**	**0.587** **P** ** < 0.001**	−0.308 *P* = 0.068	**0.983** **P** ** < 0.001**	1			
*E*_*H*_-AOB	0.063 *P* = 0.714	−0.171 *P* = 0.320	0.030 *P* = 0.861	0.087 *P* = 0.614	0.115 *P* = 0.505	−0.134 *P* = 0.434	0.305 *P* = 0.070	0.147 *P* = 0.391	1		
PC1	**0.867** **P** ** < 0.001**	**−0.577** **P** ** < 0.001**	**0.859** **P** ** < 0.001**	**0.870** **P** ** < 0.001**	**0.929** **P** ** < 0.001**	**−0.549** **P** ** < 0.001**	**0.727** **P** ** < 0.001**	**0.688** **P** ** < 0.001**	0.200 *P* = 0.241	1	
PC2	**−0.379** **P** ** = 0.023**	**0.732** **P** ** < 0.001**	−0.266 *P* = 0.117	0.052 *P* = 0.764	0.003 *P* = 0.987	0.104 *P* = 0.546	**0.659** **P** ** < 0.001**	**0.684** **P** ** < 0.001**	0.193 *P* = 0.259	0.000 *P* = 1.000	1

AOA: ammonia-oxidizing archaea; AOB: ammonia-oxidizing bacteria; *H:* Shannon-Wiener index; *S*: richness; *E*_*H*_: evenness. The effects in bold are significant at *P* < 0.05.
